# Residual Ammonium Persulfate in Nanoparticles Has Cytotoxic Effects on Cells through Epithelial-Mesenchymal Transition

**DOI:** 10.1038/s41598-017-12328-0

**Published:** 2017-09-18

**Authors:** Chen Song, Leyu Wang, Genlan Ye, Xiaoping Song, Yutong He, Xiaozhong Qiu

**Affiliations:** Deparment of Anatomy, Guangdong Provincial Key Laboratory of Construction and Detection in Tissue Engineering, Southern Medical University, Guangdong, Guangzhou 510515 China

## Abstract

Ammonium persulfate (APS), a low molecular weight chemical compound with strong oxidizing properties, should to be totally removed during preparation of nanomaterials due to its cytotoxicity. APS exerts its oxidative stress effects mainly on cell membrane, but its intracellular influence remains unclear. Here, we designed a facile negatively-charged carboxylic gelatin-methyacrylate (carbox-GelMA) nanoparticle (NP) as a cargo-carrier through the catalytic and oxidizing action of APS in W/O system. The formed APS-loaded carbox-GelMA NPs (APS/NPs) were transported into the lysosome in MCF-7 breast cancer cells. The intracellular APS/NPs produced a high level of oxidative stress in lysosome and induced epithelial-mesenchymal transition (EMT). Consequently, the MCF-7 cells challenged with APS/NPs had a strong metastatic and invasive capability *in vitro* and *in vivo*. This study highlights that a facile APS-loaded nanocarrier has cyctotoxicity on cells through EMT. Unexpectedly, we found a novel pathway inducing EMT via lysosomal oxidative stress.

## Introduction

Persulfate salts, e.g. ammonium persulfate (APS), a category of low molecular weight chemical compounds with strong oxidizing properties, are widely applied to various manufacturing processes, including the preparation of nanomaterials^1^. These compounds have been identified as a key cause of immunological sensitization and subsequent allergic diseases such as asthma, contact dermatitis, contact urticaria and anaphylaxis^2–5^. APS exerts its effect mainly outside the cell and acts on membrane proteins on cell surface, which usually give rise to a decrease in cell viability and a increase in cell apoptosis^6,7^. Thus, during the preparation of nanomaterials, APS is advised to be totally removed due to its cytotoxicity. The intracellular influence of APS remains unclear, however, when they are transforted into the cells by nanocarriers.

Epithelial-mesenchymal transition (EMT), a biologic phenotype-changed process of polarized epithelial cells, is characterized by the loss of typical epithelial markers, the acquisition of the mesenchymal properties, the enhanced migratory ability and invasiveness of tissues^8^. EMT plays important roles during animal embryonic development. In adults, EMT occurs abnormally, and results in the tumor progression^9,10^. The activation of EMT has a close relationship with the cancer progression and cancer matastasis^11–13^. The full spectrum of signaling agents that contribute to EMT of carcinoma cells remains unclear, although many efforts are invested on it. In order to study the mechanism of EMT, the epithelial cancer cells were usually transformed into the mesenchymal-like cells *in vitro* through the prolonged mammosphere culture^14^, or the gene transfer technology etc^15^. However, most of these evoked strategies for EMT are expensive and time-consuming. The three-dimensional multicellular tumor spheroid was employed in our previous study to simulate a tumor microenvironment and was found to trigger EMT in breast cancer cells^16^, while the fabrication procedure of this model was relatively complicated. Similarly, many other studies mainly utilized spatial architecture built with biomaterials to regulate EMT of cells^17,18^. Whereas, there are few ways utilizing nanotechnology to directly develop the EMT-related cell model.

**Figure 1:**
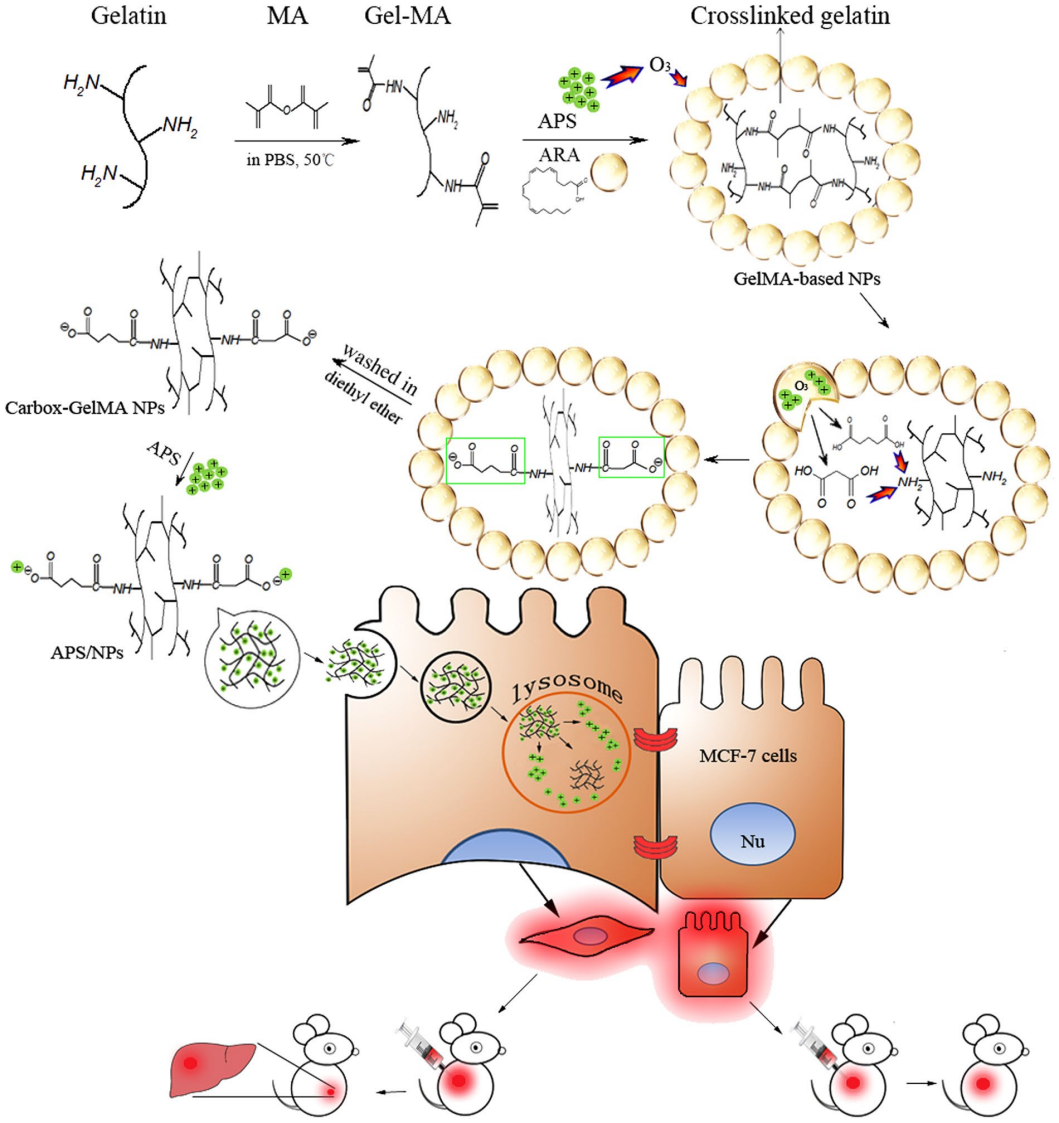
The scheme describing the fabrication process of APS-loaded carbox-GelMA nanoparticles (APS/NPs) and their influence on EMT in breast cancer MCF-7 cells. ARA and GelMA were emulsively blending to form the water-in-oil (W/O) mixture, and then the mixture was catalyzed to form GelMA-based NPs by APS. Meanwhile, ARA was oxidized to produce malonic acid and glutaric acid by APS. The carboxyl groups in malonic acid and glutaric acid could react with the partial free amino group in GelMA to generate the negative charges. After the excessive oil layer was washed by diethyl ether, the carbox-GelMA NPs were produced. The negatively-charged carbox-GelMA NPs could carry the positively-charged APS through electrostatic interaction. The APS/NPs showed high effective induction for EMT in MCF-7 cells *in vitro* through lysosome pathway and endow MCF-7 cells the metastasic capability to liver *in vivo*.

In this study, a facile APS-loaded carboxylic methyacrylate gelatin (carbox-GelMA) nanoparticle (NP) was designed to induce the EMT. Gelatin, as a nature emulsifier, is a hydrolytic production derived from collagen with its low immunogenicity and good biocompatibility, and has been widely employed in drug delivery, gene therapy and tissue engineering^19,20^. The gelatin microspheres or NPs could be produced through W/O emulsion methods^21,22^. Here, the gelatin-methyacrylate (GelMA), which contained the structure of gelatin and double bond resulting from the free amino group in gelatin conjugated with methyacrylate^20^, was applied as the emulsifier. The structure of double bond in GelMA endows it the capability of self-crosslinking. Arachidonic acid (ARA) is a sort of polyunsaturated fatty acid, and its analogue, such as oleic acid and docosahexaenoic acid, have been used in the preparation of lipid-polymer nanomaterials^23,24^. As shown in Fig. 1, according to our design, the GelMA, ARA and APS were simultaneously introduced into a W/O system to form the GelMA-based NPs. Under the W/O system, GelMA is catalyzed by APS into self-crosslinked GelMA mesh^25^, meanwhile ARA in W/O system is oxidized by APS to yield malonic acid and glutaric acid^26^. Both of malonic acid and glutaric acid react with amino groups on GelMA-based NPs, endow the NPs with the negatively charged carboxylic groups, and form the carboxylic GelMA (carbox-GelMA) NPs. The functional carbox-GelMA NPs with the negative charges are appropriate carriers for the positively-charged APS.

Oxidative stress was confirmed to promote epithelial to mesenchymal transition, while APS is always served as a strong oxidant in the synthesis of biomaterials^27,28^. Therefore, we hypothesize that the APS-loaded carbox-GelMA NPs (APS/NPs) induce epithelial-mesenchymal transition (EMT). To test this hypothesis, we treated MCF-7 cells with APS/NPs, we found that the APS/NPs-induced aggressive MCF-7 (MCF-7-EMT) cells have a strong migratory and invasive ability both *in vitro* and *in vivo*. Furthermore, the mechanism underlying APS/NPs-induced EMT was explored.

## Results

### Characterizations of carbox-GelMA NPs

**Figure 2:**
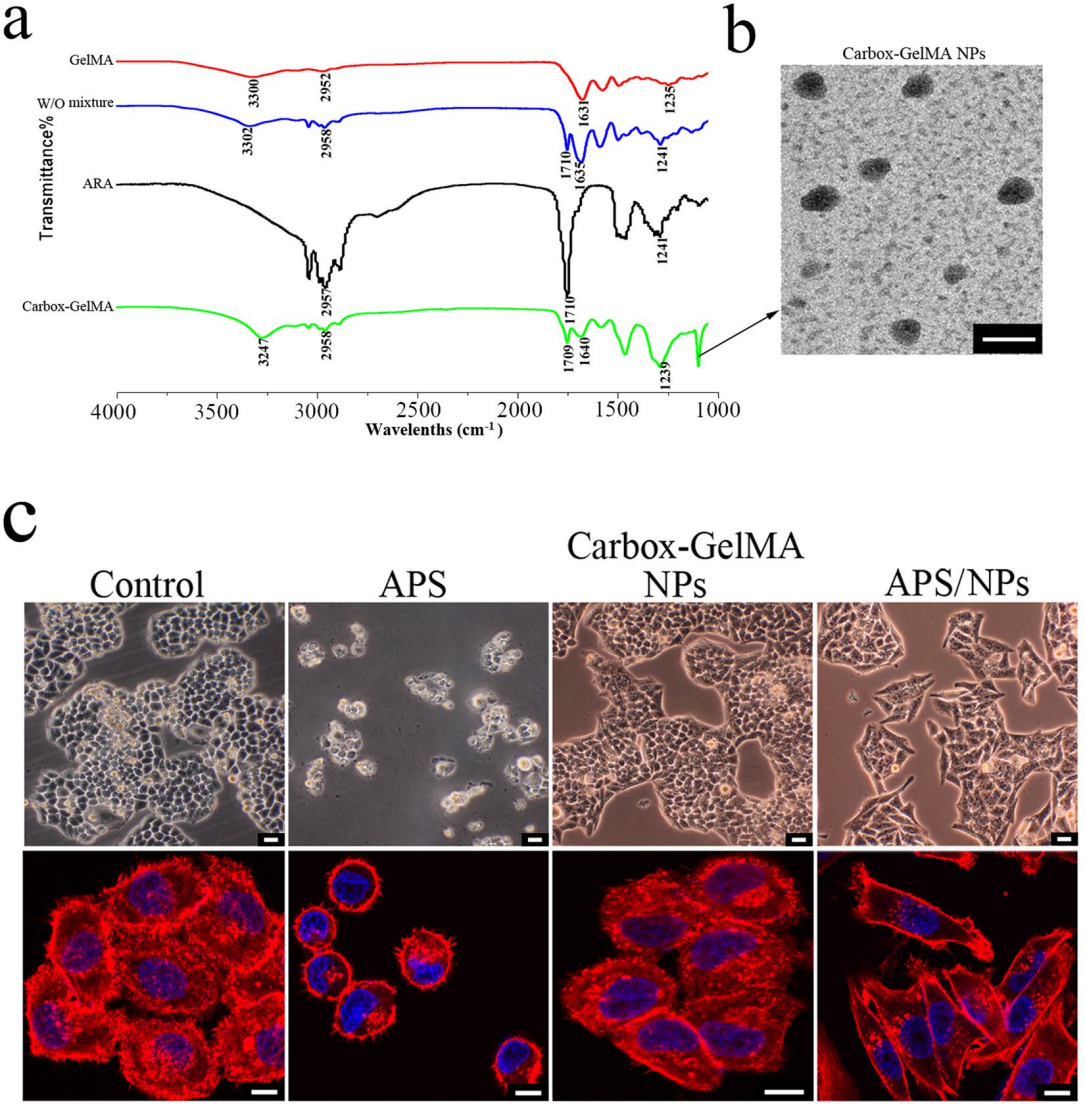
Characterization of carbox-GelMA NPs and their influence for MCF-7 breast cancer cells. (**a**) The component analysis of ARA, GelMA, W/O GelMA/ARA mixture (W/O mixture) and carbox-GelMA NPs by the Fourier transform infrared spectroscopy (FTIR). (**b**) The ultrastructure of carbox-GelMA NPs under transmission electron microscopy (TEM). Scale bars: 200 nm. (**c**) The upper row: The MCF-7 cells shapes were observed under phase-contrast microscopy. Scale bars: 100 µm. The lower row: The F-actin staining MCF-7 cells were observed under confocal microscope. Scale bars: 20 µm.

The vibrational/adsorption bands of different bonds in the prepared samples were analyzed through Fourier transform infrared spectroscopy(FTIR). The FTIR spectra of W/O GelMA/ARA mixture included the characteristic bands both in GelMA IR pattern (the wide stretching vibration bands of O-H or N-H bands around 3290 cm^−1^ and the C=O stretching vibration bonds around 1700 cm^−1^)^22^ and in ARA IR pattern (the C-H stretching vibration bonds in carbon chains around 2900 cm^−1^ and the C=C stretching vibration bonds around 1630 cm^−1^), suggesting that the emulsive W/O GelMA/ARA mixtures were consisted of ARA and GelMA (Fig. 2a). Reinforcement of C-O stretching vibration bonds (around 1200 cm^−1^) and C=O stretching vibration bonds (around 1700 cm^−1^) were more obviously found in the carbox-GelMA NPs than those in the W/O GelMA/ARA mixture, mainly due to the acquirement of carboxyl group from the oxidized production of ARA by APS compounds (Fig. 2a). Under TEM microscope, most of the carbox-GelMA NPs were spherical and well-seperated (Fig. 2b) because of the existent repulsion between particles^29^. The size of produced carbox-GelMA NPs were varied from 100 nm to 300 nm, with a mean of 182.5 nm (Figure S1a). Meanwhile, the average zeta potential of carbox-GelMA NPs was −14.02 ± 0.97 mv resulting from the reactions between the oxidative products of ARA (malonic acid or glutaric acid) and the amino groups in GelMA (Figure S1b). While, the average charge in carbox-GelMA NPs was increased to −0.18 ± 0.06 mv after the APS compounds were loaded onto the carbox-GelMA NPs (Figure S1b). Additionally, the pH value of the solution containing pure carbox-GelMA NPs was 6.86, and the pH value of the solution containing APS/NPs was 7.02.

### APS/NPs could activate EMT progress in breast cancer MCF-7 cells *in vitro*

Different cell fates of MCF-7 breast cancer cells could be triggered by different APS treatment ways (pure APS or APS/NPs). The typical clustered epithelial-like cells with tight cell-cell connections were displayed in the pure carbox-GelMA NPs group and in the control group, while the scattered apoptotic-like round cells were displayed in the pure APS group (Fig. 2c, upper row). Interestingly, lots of spindle shape cells were present in the APS/NPs group (Fig. 2c, upper row). The cell morphology displayed by F-actin-marked cytoskeleton confirmed the changes of MCF-7 cells from epithelial-like morphology (compactly arranged morphology) in the control group to mesenchyme-like morphology (scattered spindle morphology) in the APS/NPs group (Fig. 2c, lower row). MCF-7 cell is a luminal-like breast cancer cell line bearing epithelial morphology and is usually taken as an experimental cell model to study EMT progress of breast cancer *in vitro* and *in vivo* ^30,31^. In this study, the changes in cellular morphology in the APS/NPs-treated cells suggest that they are undergoing EMT. The cell viability in each group was further investigated by CCK-8 assay. As that in untreated cells (control group), a high cell viability was kept in carbox-GelMA NPs-treated group. The cells viability of MCF-7 cells was decreased in a time dependent manner in the pure APS-treated group. While, the cell viability in the APS/NPs-treated group was decreased gradually from 12 hrs to 48 hrs, then it reached to a stable level from 48 hrs to 96 hrs (Figure S2). The cells treated with APS/NPs for 72 hrs (MCF-7-EMT cells) were collected. They became mesenchymal-like spindle shape. The survived MCF-7-EMT cells were amplified in RPMI 1640 medium and used for futher analysis with fluorescence activating cell sorter (FACS) analysis, wound healing assessment and animal experiments.

**Figure 3:**
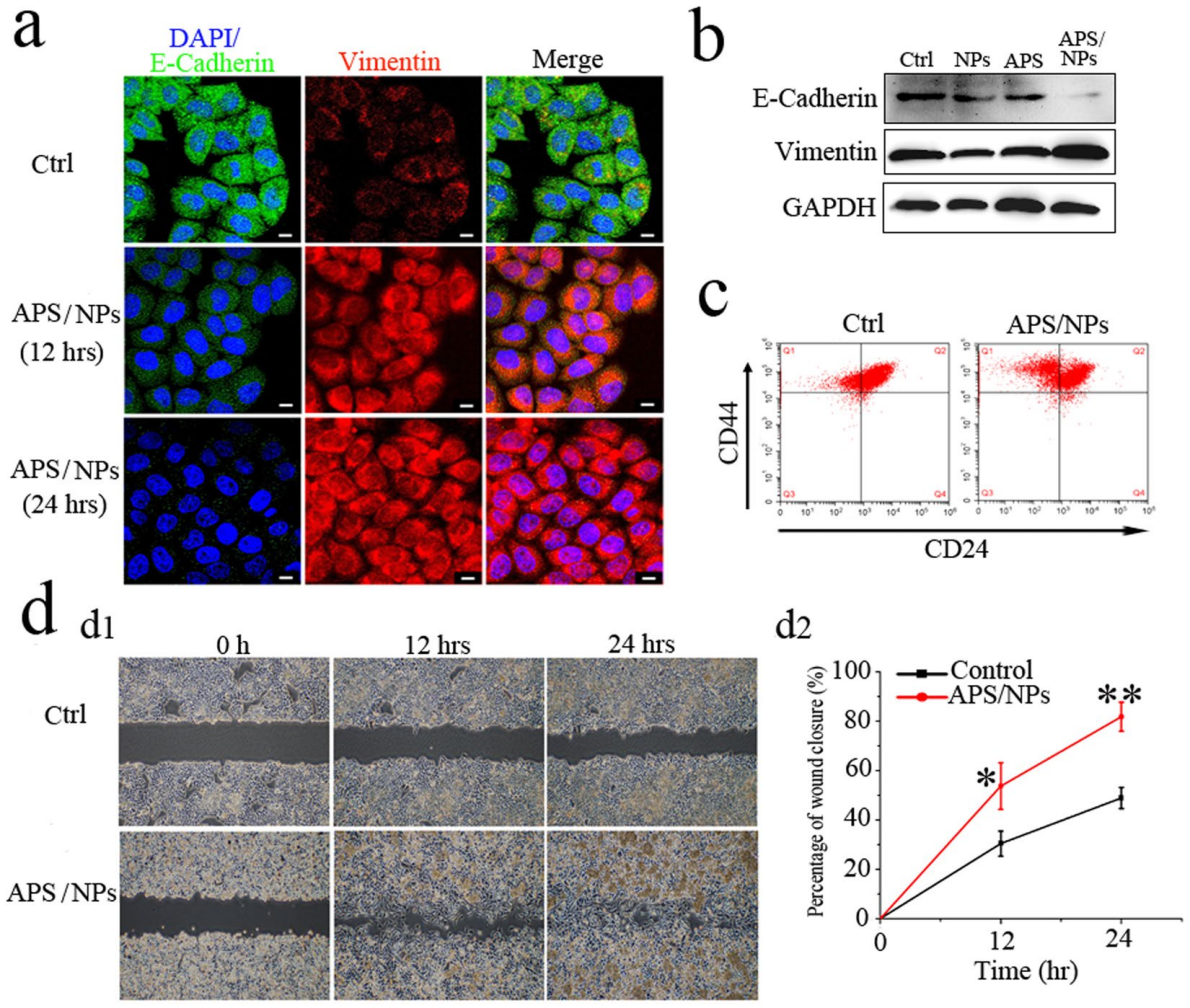
APS/NPs could activate EMT, increase the migration ability and the stemness in breast cancer MCF-7 cell lines *in vitro*. (**a**) The expression of epithelial marker E-Cadherin (green) and mesenchymal marker vimentin (red) in MCF-7 cells was detected by immunoflurescence staining. The expression of E-cadherin was downregulated whereas the expression of vimentin was upregulated in the MCF-7 cells treated with APS/NPs for 12 hrs and 24 hrs, compared to the control group (Ctrl, untreated cells). Nuclei were stained with DAPI (blue). Scale bars: 10 μm. (**b**) The expressions of E-Cadherin protein and vimentin protein in MCF-7 cells were detected by western blotting. The MCF-7 cells were treated with pure carbox-GelMA nanoparticles (NPs), pure APS (APS) and APS/NPs for 24 hrs respectively, the untreated cells were taken as the control group. Low expression of E-cadherin protein whereas high expression of vimentin protein appeared simultaneously in APS/NPs group. (**c**) FACS analysis of cell-surface markers, CD44 and CD24, in MCF-7-EMT cells. The MCF-7 cells being treated with APS/NPs for 72 hrs were named MCF-7-EMT cells (see text part). More CD44^high^/CD24^low^cells were detected in MCF-7-EMT cells, compared with the untreated cells. (**d**) Wound-healing assay of cell migration capability. The MCF-7-EMT cells exhibited significantly higher migration rates, compared with the untreated cells.

During the EMT progress, the morphology alterations are often accompanied with the changes of gene expression and cell motility^32^. The loss of E-cadherin (a typical epithelial marker) and the induction of vimentin (a typical mesenchymal marker) are vital signals of EMT progress^33,34^. As predicted, a gradual downregulation of E-cadherin and a gradual upregulation of vimentin were clearly observed using immunostaining in MCF-7 cells, which were treated with the APS/NPs from 12 hrs to 24 hrs (Fig. 3a). Meanwhile, the same results were confirmed by western blot analysis (Fig. 3b). Because EMT progress in cancer cells generates cancer stem cells^35^, two cell-surface stemness markers (CD44 and CD24) were investigated in APS/NPs-induced MCF-7-EMT cells by FACS. As shown in Fig. 3c, the MCF-7-EMT cells acquired the stem cells properties with CD44^high^/CD24^low^ antigen phenotype^36^. To test the cell motility, a wounding assay was performed. The wound closure rate (WCR) in MCF-7-EMT cells at 12 hrs and 24 hrs were 1.8 times and 1.7 times higher than those in untreated cells (control group) respectively (Fig. 3d). The similar phenomenon was observed in another breast cancer cell line MDA-MB-231, which exhibited more mesenchymal properties after challenged with the APS/NPs for 72 hrs (Figure S3). Our results indicate that the APS/NPs treatment significantly enhances the migration ability of cells.

### APS/NPs-induced MCF-7-EMT cells have high invasive and metastasis ability *in vivo*

**Figure 4:**
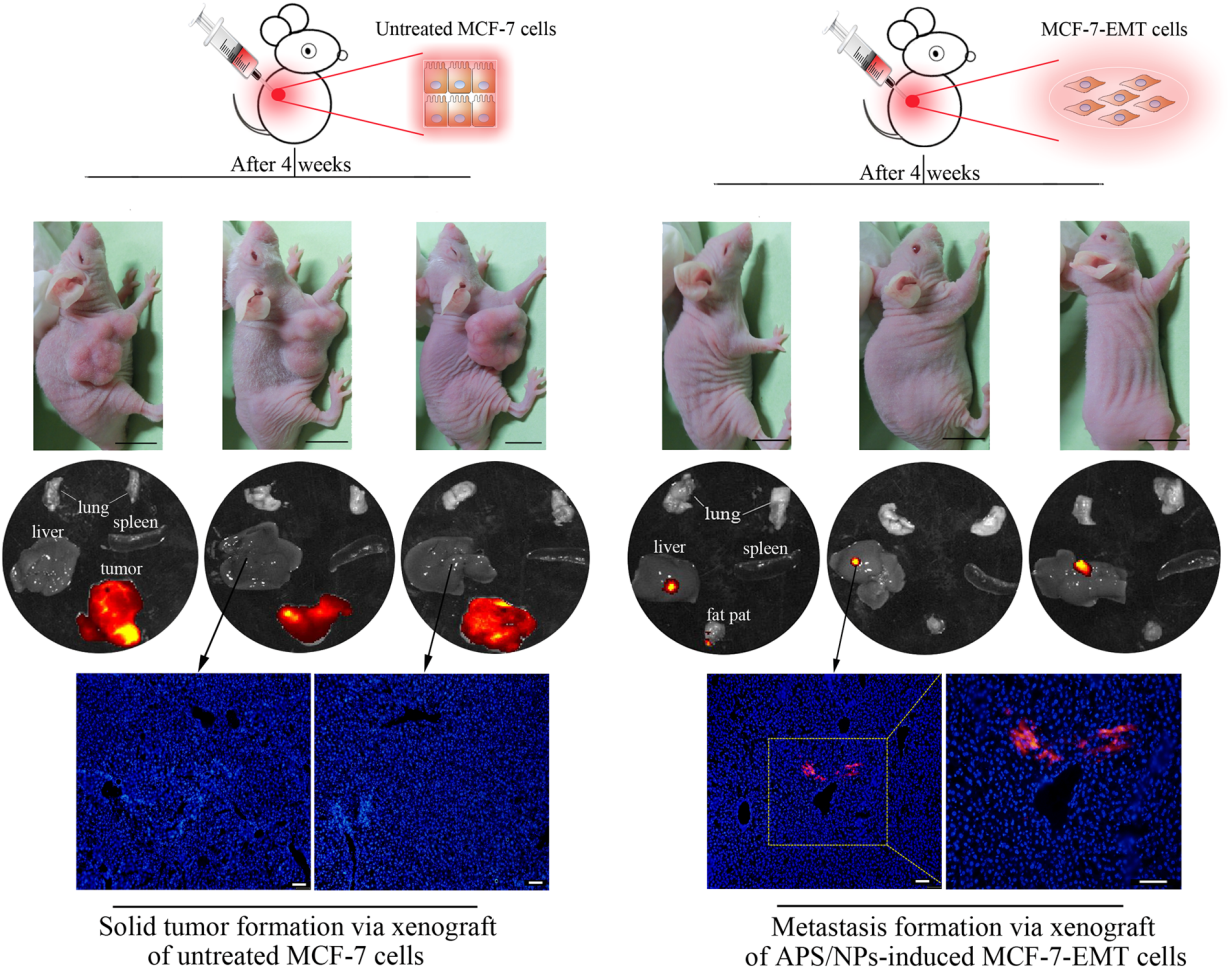
The APS/NPs-induced MCF-7-EMT cells owned strong metastatic ability *in vivo*. The DiI+ untreated MCF-7 cells and the DiI+ APS/NPs-induced MCF-7-EMT cells were orthotopically transplanted into the BALB/c nude mice respectively. 4 weeks later, solid tumors were obviously found in the untreated MCF-7 cells-transplantation mice, while no evident solid tumors were found in the MCF-7-EMT cells-transplanted mice. The fluorescent signals of the major organs, including the lungs, livers, spleens and tumor tissues (or fat pats) from the mice in 2 groups were detected. The DiI+ signals (red) were gathered in tumor tissues in the untreated MCF-7 cells-transplanted mice, while the DiI+ signals were detected in the livers of the MCF-7-EMT cells-transplanted mice. The fluorescence analysis of liver tissue sections further revealed that the DiI-marked cells had migrated into the liver tissues in the MCF-7-EMT cells-transplanted group. Blue: DAPI-stained nucleus. Black scale bars: 1 cm; White scale bars: 100 µm.

Accumulating evidence suggests that the EMT could promote the invasive and metastasis ability in breast cancer cells^11,37^. To evaluate the aggressive capability of MCF-7-EMT cells in vivo, the celltracker CM-DiI pre-stained MCF-7-EMT cells were orthotopically transplanted into the nude mice. The mice orthotopically transplanted with CM-DiI pre-stained untreated MCF-7 cells were taken as the control group. Four weeks later, none of the 6 MCF-7-EMT cells-transplanted mice had solid tumors while all of 6 control mice displayed obviously solid tumors. The evident DiI^+^ signals were detected in livers in MCF-7-EMT cells-transplanted mice using fluorescent signals detection *ex vivo* organs (Fig. 4). Collectively, our results suggest that the MCF-7-EMT cells possess highly aggressive capability. The breast cancer cells with a higher invasive ability produced a bigger solid tumor volume than those with a lower invasive ability after they were subcutaneous transplanted into nude mice^13,38^. Curiously, in this study, the APS/NPs-triggered MCF-7-EMT cells developed no macroscopic tumors but gained a high incidence rate (>80%) of liver metastasis. As a reasonable explanation, metastatic cancer is a highly heterogeneous disease and the metastatic solid tumors derived from different individuals had a different representation at genetic and transcriptomic levels^39^. Thus, in this study, the cell fate of the exogenous mesenchymal-liked cells screened by APS/NPs is complicated and unpredictable *in vivo*. The large sample data, which were used to investigate the integrative clinical genomics in metastatic cancers, showed that there were about 5 percent carcinomas whose primary origins were unclear^40^. The formation of solid tumor in the inoculation site is partly dependent on the cancer cell numbers. Herein, the MCF-7-EMT cells, which were used for subcutaneously transplantation into mice, had the stable and high cell viability and the strong metastatic ability. Most of the transplanted MCF-7-EMT cells might metastasize in a short time, and the number of locally remaining cells are not sufficient to form a solid tumor.

### The oxidative stress of lysosome triggered by APS/NPs could induce EMT

**Figure 5:**
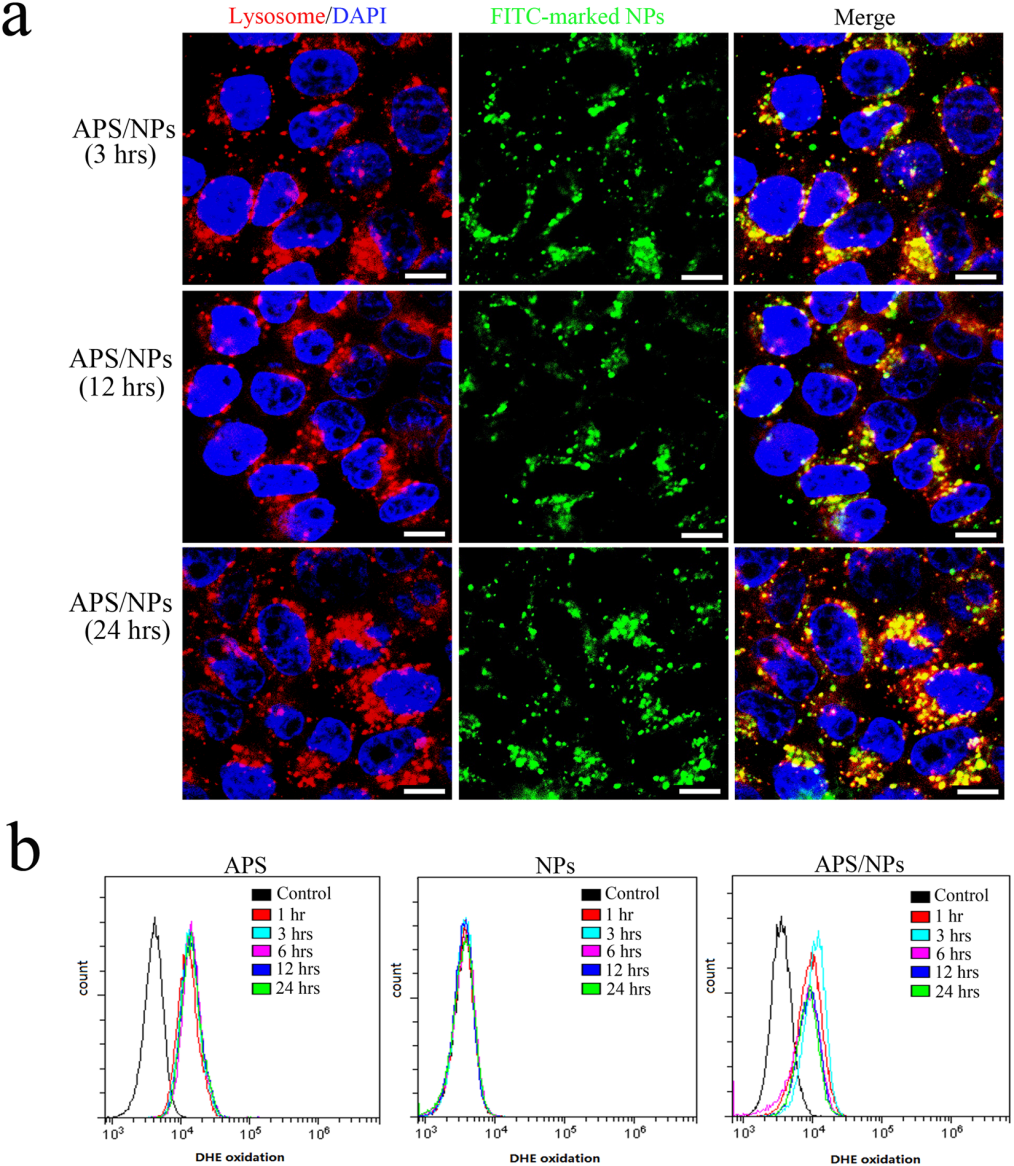
The oxidant stress in lysosome induced by APS could activate EMT in MCF-7 cells. (**a**) Fluorescence co-localization of lysosome (red) and FITC-labeled NPs (green) after the MCF-7 cells were treated with APS/NPs for 3 hrs, 12 hrs, and 24 hrs respectively. Yellow: the overlap views of lysosome with NPs. Blue: DAPI-stained nucleus. The scale bars: 10 µm. (**b**) FACS analysis for intracellular ROS levels in MCF-7 cells after the cells were treated with APS, NPs, and APS/NPs respectively for different times.

Our results suggest that APS/NPs could induce EMT in MCF-7 cells. Therefore, we asked how the APS/NPs exert their influence on inducing EMT. A mechanistic understanding about how the EMT is induced remains insufficient, although EMT has a certain effect on cancer progression and cancer metastasis. We speculated that APS/NPs could be uptaken by lysosome, and then promote EMT progress via a high level of oxidative stress in lysosome. To test this possibility, the fluorescent signals both of FITC-labeled carbox-GelMA NPs (green) and of lysotracker-labeled lysosome (red) in MCF-7 cells were detected in different groups by CLSM. The pure carbox-GelMA NPs could easily enter into the lysosome, and the obviously yellow overlaid areas were detected in the carbox-GelMA NPs-treated cells (Figure S4). Furthermore, as shown in Fig. 5a, the apparent colocalization of APS/NPs-FITC with lysotracker-labeled lysosome was observed, indicating that carbox-GelMA NPs carry APS into lysosome. The pure NPs doesn’t induce EMT, which indicates the NPs entry into lysosome alone is insufficient for inducing EMT. Thus, we hypothesized that a high level of oxidative stress in lysosome triggered by APS could induce EMT progress. Therefore, the cellular reactive oxygen species (ROS) levels were detected by FACS using specific ROS probes. As shown in Fig. 5b, both APS and APS/NPs treatments could significantly increase the cellular ROS level in the first hour and the high level of ROS could sustain for 24 hrs, while the carbox-GelMA NPs alone had no evident effects on increasing the cellular ROS level. As a strong oxidant, APS induces EMT in MCF-7 cells only when it enters lysosome, then triggers oxidative stress in lysosome. Otherwise, APS alone only exerts its effect mainly outside of cells and works on membrane proteins on cell surface^7^. It could be speculated that after APS/NPs were endocytosed into lysosomes, the charge of carbox-GelMA NPs was conversed under the acidic environments of lysosome^41^, and the APS compounds were released. The vast oxidative stress in lysosome, triggered by APS, would induce EMT in breast cancer cells.

### Distinct gene expression profiles among the untreated MCF-7 cells, APS-treated MCF-7 cells and APS/NPs-induced MCF-7-EMT cells

**Figure 6:**
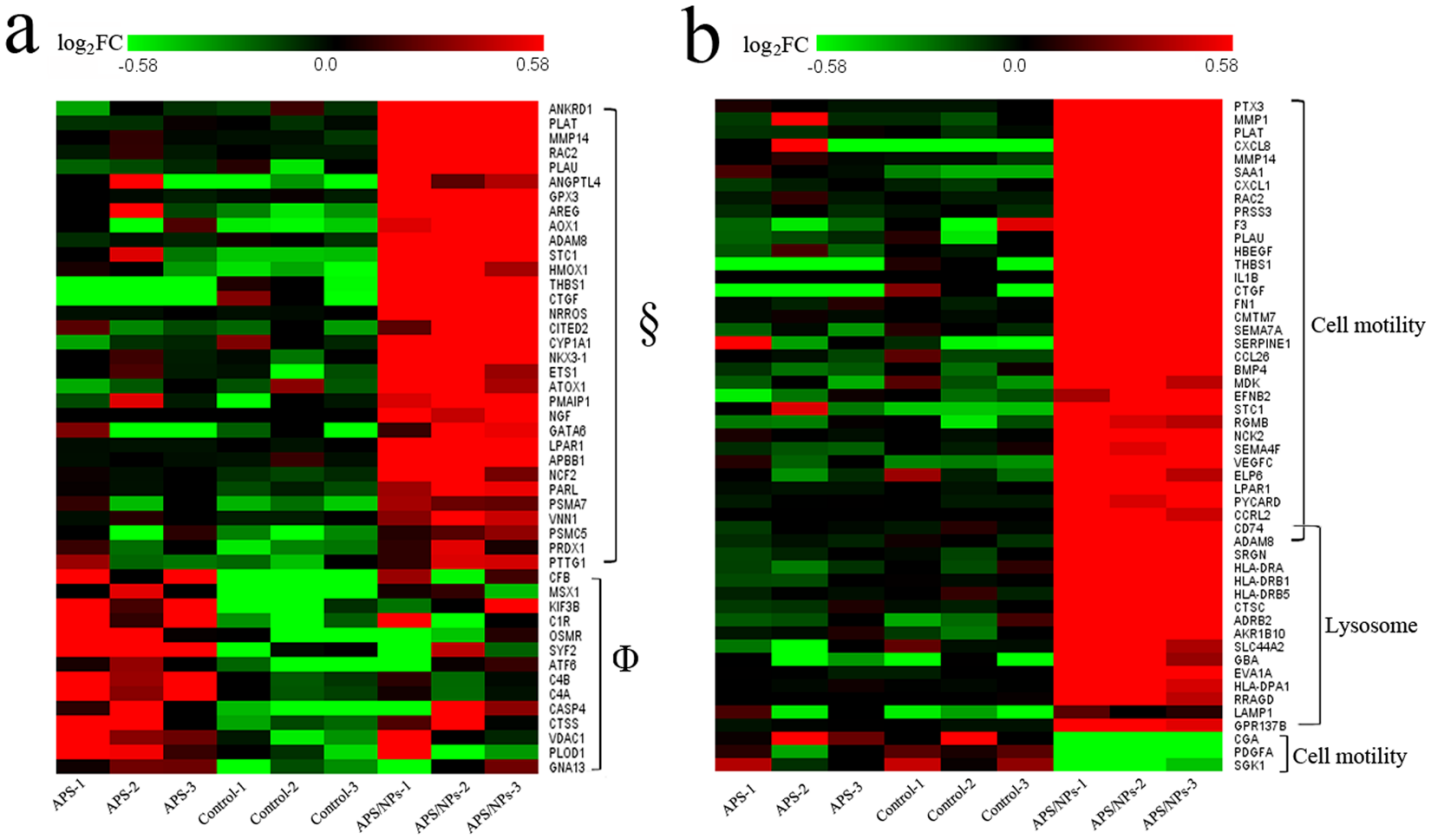
The heatmap of differently expressed genes among the APS-treated MCF-7 cells, the APS/NPs-induced MCF-7-EMT cells, and the untreated MCF-7 cells. (**a**) The heatmap of oxidative stress-related genes in the APS-treated MCF-7 cells (APS groups), the APS/NPs-induced MCF-7-EMT cells (APS/NPs groups), and the untreated MCF-7 cells (control groups). ^§^Means the differently expressed genes between the ANP/NPs groups and the control groups. Φ means the differently expressed genes between the APS groups and the control groups. (**b**) The heatmap of cell motility-related and lysosome-related genes among the APS-treated MCF-7 cells (APS groups), the APS/NPs-induced MCF-7-EMT cells (APS/NPs groups), and the untreated MCF-7 cells (control groups). Green area means downregulated expression and red area means upregulated expression.

To comprehensively study the cellular pathways of oxidative stress induced by APS/NPs and by the pure APS compounds, cDNA samples of the untreated MCF-7 cells (n = 3), the pure APS-treated MCF-7 cells (n = 3) and the MCF-7-EMT cells (n = 3) were prepared for RNA-seq analysis. The heatmap of differential oxidative stress-related genes were shown in Fig. 6a, 24 oxidative stress-related genes were high expressed in the MCF-7-EMT cells (APS/NPs groups) compared to the pure APS-treated cells (APS groups). Among them, 9 genes (ANKRD1, PLAT, MMP14, RAC2, PLAU, THBS1, CTGF, GPX3 and ADAM8) had more than 3 fold changes in the APS/NPs groups compared to the APS groups. The different levels of oxidative stress-related mRNAs caused different consequences. As shown in Fig. 6b, 37 cell motility-related genes were differentially expressed (34 genes up-regulated, and 3 genes down-regulated) in the APS/NPs groups compared to the APS groups. Among them, 11 genes (BMP4^42^, STC1^43^, SERPINE1^44^, CXCL8^44,45^, CXCL1^45^, EFNB2^46^, MMP14^47^, VEGFC^48^, CCRL2^49^, FN1^50^ and MMP1^47^) have close relationship with EMT. However, there is no different expression of the cell motility-related genes between the APS groups and the control groups. As for the lysosome-related genes, 16 differential expressed genes, including the lysosome membrane marker, LAMP1, were highly expressed in the APS/NPs groups, but low both in the APS groups and in the control groups. These results provided a molecular evidence for APS/NPs to induce EMT in breast cancer cells. These data support that the EMT in breast cancer cells could be induced by the high level of oxidative stress in lysosome. More interestingly, the MCF-7-EMT cells also possessed a larger amount of up-regulated immune response-related genes, including MHC (major histocompatibility complex) II genes, than the pure APS-treated cells and the untreated cells, indicating that MCF-7-EMT cells are immune-edited during their EMT progress and might be a risk of immunological recognition *in vivo* (Figure S5). This phenomenon is consistent with the gene expression profiling in the aggressive MCF-7 cells derived from the coculture of MCF-7 cells with osteosarcoma cells, which has 42% differential gene belonging to immune answer^31^.

## Discussion

In this study, we fabricated a facile carbox-GelMA nanoparticle based on the W/O emulsion methods. Once the APS is loaded onto the carbox-GelMA NPs, the formed APS/NPs are transported into the lysosome in MCF-7 cells, and the high level of ROS in lysosome induce EMT. The active lysosome, evoked with EMT-related inducers, could rearrange the cytoskeleton and facilitate the acquisition of invasion ability in the malignant mammary epithelial cells^51,52^. While, the mechanism about lysosome-induced EMTs are insufficient except a litter views were proposed, which including the ubiquitination and degradation of E-cadherin, or the contribution of lysosomal protease for the invasion ability of the transitional epithelial cells^52–54^. The results in this study could be recapitulated as a potent intracellular switch of APS oxidant for activating lysosome to perform EMT progress. Relying on a facile negative carbox-GelMA NP, different biological effects of the same oxidant in different area of cell were achieved. The ROS, key signaling molecules during homeostasis and cell signaling^55,56^, are involved in initiation, promotion, and malignant progression for cancers of the breast, liver, and colon^56^. It is proved that ROS play an important role in promoting EMTs^56–58^, and EMTs could be introduced by increasing ROS production directly^59^ or indirectly through downregulation of antioxidant system^60^. As for our results, a high level of ROS in cytomembrane mainly lead to cell apoptosis and necrosis, while a high level of ROS in lysosome would lead to cell EMT progress.

Metastasis, a hallmark of malignancy, is a crucial process during cancer progression. EMTs could endow carcinoma cells with invasive and metastatic abilities. Thus, understanding of its molecular mechanisms could help us to identify new key therapeutic targets for cancer. Our results suggest that APS/NPs enter lysosome and release APS, and then induce a high level of oxidative stress and EMTs. ROS result in lipid peroxidation, DNA damage and activation of signaling events, which are associated with a loss of cell growth, carcinogenesis and fibrosis^61–63^. ROS can be served as the intracelluar mediators of the TGF-β1-induced EMT^64^. Repeated exposure of cells to MMP-3 resulted in loss of E-cadherin, nuclear transcription of β-catenin, activation of TGF/LEF transcriptional activity, the common features of EMT^65^. More precisely, MMP-3 increases the generation of ROS, eventually leads to up-regulation of SNAIL, and then induces EMT^65,66^. In this study, both APS and APS/NPs treatment could significantly increase cellular ROS level. A high level of ROS in cell plasma membrane mainly leads to cell apoptosis and necrosis, but a high level of ROS in lysosome lead to cell EMT progress. Also, the EMT induced by APS/NPs in cellular lysosome was also proved through the differential expression of oxidative stress-related genes, cell motility-related genes, lysosome-related genes and immune response-related genes in the APS/NPs-challenged MCF-7 breast cancer cells. The diverse cell fates, death or transition, could be arrived by the same dose of APS oxidants in different cellular areas. Residual APS in nanoparticles can trigger EMT through lysosome pathway in breast cancer cells. Based on this evidence, residual APS should be completely removed when it is applied in the synthesis of biomaterials.

## Methods

### Preparation of nanoparticles (NPs)

The synthetic method and characterization of methacrylated gelatin (GelMA) were described in our previous paper^20^. Briefly, 0.375% (w/v) GelMA was completely dissolved in ddH2O at 50 °C. Then 0.375% (w/v) arachidonic acid (ARA, Aladdin, China) and 0.25% (w/v) ammonium persulfate (APS, Sigma-Aldrich, USA) were sequentially introduced to the GelMA solution for reacting overnight at room temperature under sufficiently stirring. The mixture was lyophilized, washed by diethyl ether to discard the additional ARA, and dried under soft conditions (room temperature and atmospheric pressure). The carbox-GelMA NPs were obtained by dialysis against deionized water and lyophilization. As to the assembly of APS-loaded carbox-GelMA NPs (APS/NPs), 0.3 mg APS was added into 100 µl PBS containing 0.5 mg carbox-GelMA NPs and was shaken softly at 4 °C overnight. The mixture was then centrifuged at 10000 g at 4 °C for 15 min and the supernatant was collected as the APS/NPs stock solution.

### Characterization of the carbox-GelMA NPs

Fourier Transform Infrared (FTIR) spectroscopy was used to determine the chemical bonding and composition of the samples. The dried samples were ground with KBr powder in an agate mortar and compressed into pellets for FTIR examination; while the ARA sample was uniformly coated on the surface of KBr wafer for FTIR examination. FTIR spectra were then recorded with a Thermo Scientific IR6700 FT-IR Spectrometer. The morphology of NPs were observed by Transmission Electron Microscopy (TEM) (Tecnai^TM^ G2 Spirit, FEI, USA). The size distrubutions and the zeta-potential of NPs were evaluated by Dynamic Light Scattering (DLS) (Zetasizer Nano-Zs, Malvern Instruments, UK).

### Cell culture

The breast cancer cell lines,MCF-7 and MDA-MB-231, were purchased from ATCC (USA). MCF-7 cells were cultured in RPMI 1640 medium (GIBCO) supplemented with 10% fetal bovine serum (FBS, GIBCO), 100 U/ml penicillin, and 100 μg/ml streptomycin. MDA-MB-231 cells were maintained in high-glucose Dulbecco’s modified Eagle’s medium (DMEM) medium supplemented with 10% fetal bovine serum (FBS, GIBCO), 100 U/ml penicillin, and 100 μg/ml streptomycin. Cells were maintained in an incubator at 37 °C under 5% CO_2_ condition and the mediums were exchanged every 2 days. In order to explore the influence of APS/NPs on the cells, the cells were treated by APS/NPs (containing 0.3 mg/ml APS and 0.5 mg/ml carbox-GelMA NPs), 0.3 mg/ml pure APS and 0.5 mg/ml carbox-GelMA NPs respectively. The untreated cells were taken as the control group.

### Confocal microscope observation

Confocal laser scanning microscope (LSM 880, Zeiss, Germany) was employed for the observation of the cytoskeleton through F-actin staining, the expressions of E-cadherin and vimentin, and the colocalization of NPs with lysosome in cells. The fixed cells were permeabilized with Triton X-100 for F-actin staining with rhodamine phalloidin (Vigorous) as described previously^37^. As for the detection of protein levels, the fixed cells were permeabilized with Triton X-100, blocked with 2% bovine serum albumin (BSA) in PBS at room temperature for 30 min, and incubated with Rabbit anti-E-cadherin antibody (1:25) (Abcam, UK) plus Mouse anti-Vimentin antibody (1:100) (Abcam, UK) at 4 °C for overnight. After being washed with PBS for three times, cells were incubated in Alexa Fluor488 Donkey Anti-Rabbit IgG (H&L) (1:500) and Alexa Fluor568 Donkey Anti-Mouse IgG (H&L) (1:500) for 1 hr in darkness. As for the colocalization observation, cells challenged with APS/FITC-labeled NPs were stained by 50 nM LysoTracker Red (Life Techologies) for 1 hr at 37 °C according to the supplier’s protocol. All of the samples were further stained with DAPI and imaged under confocal microscope.

### Western blot analysis

Cells were lysed using radio-immunoprecipitation assay (RIPA) protein extraction solution (KeyGEN, China). Total protein concentrations were detected by BCA Protein Assay (KeyGEN, China). Equal quantities of protein (10 µg/lane) were separated by electrophoresis on 10% SDS-PAGE and then transferred onto polyvinylidene difluoride (PVDF) membranes. The membranes were blocked for 2 hrs in TBS-T buffer (10 mmol/L Tris·HCl, pH 7.5, 500 mmol/L NaCl, 0.05% Tween 20) skim milk at room temperature and then incubated overnight at 4 °C with primary antibodies: anti-E-cadherin(1:250), anti-Vimentin(1:1000), anti-GADPH(1:3000). After being washed with TBS-T buffer three times, the membranes were incubated with the appropriate horseradish peroxidase (HRP)-linked secondary antibodies for 1 hr at room temperature. Proteins were visualized using a Supersignal Chemiluminescent substrate (Santa Cruz) according to the manufacturer’s instructions. GADPH was used as an internal standard.

### FACS analysis

The ratio of CD44^high^/CD24^low^ cells and the ROS levels in MCF-7 cells in different groups were measured by FACS analysis (Coulter Cytoflex, Beckman, USA). As for the measurement of the ratio of CD44^high^/CD24^low^ cells, cells were stained with antibodies against APC-conjugated CD24 (eBioscience, USA) and FITC-conjugated CD44 (eBioscience, USA) as described previously^35^. Cells were incubated with 10 µM dihydroethidium (DHE) at 37 °C for 1 hr in darkness for ROS levels detection, according to the supplier’s protocol.

### Wound healing assay

Cells were kept culturing in 6-well culture plates till confluent. Wounds were made by scraping with a sterilized pipette tip. After scratching, the wound healing of the cells was observed and photographed at different culturing intervals. The distances between the two edges of wounds were measured using Image J software. The wound closure rate (WCR) was calculated as following: WCR = (DS_S_ − DSc)/DSs. DS_S_ mean the distances between the two edges of wound scratching. DSc mean the distances between the two edges of wound closure.

### Animals Experiments

All of the animal experiments procedures were performed with the approval of the Southern Medical University Amimal Ethics Committee according to the Regulations for the Administration of Affairs Concerning Experimental Animals (China). Female athymic BALB/c nude mice at the age of 5–6 weeks were purchased from the Animal Center of Southern Medical University, China. The mice were randomly divided into two groups (n = 6). Before transplantation, the untreated MCF-7 cells and the APS/NPs-induced MCF-7-EMT cells were labeled with CM-DiI (2 ug/ml, Invitrogen) for tracking the implanted cells. About 6 × 10^6^ cells were subcutaneously injected into the right mammary fat pad of the mice. The mice were closely monitored, and the body weight and tumor sizes of them were measured every two days. After 4 weeks from transplantation, the mice were sacrificed and the tumors, fat pads, livers, lungs and spleens were removed. The fluorescent signals of *ex vivo* organs were evaluated by the IVIS Imaging system (Xenogen IVIS Lumina II, USA). Then the livers in two groups were fixed in 4% paraformaldehyde at 4 °C overnight, dehydrated, embedded into OCT and frozen at −80 °C. The frozen blocks were sectioned into 6 μm sections in the Leica CM1950 cryostat. The sections were stained with DAPI and images were analyzed by fluorescence microscope.

### RNA-seq analysis

Total cellular RNA was extracted using TRIzol (Invitrogen) according to the supplier’s protocol. RNA integrity was verified by agarose gel electrophoresis. RNA concentration was determined with NanoDrop ND-1000. After cDNA libraries were constructed^67^, the quality assessment of each library was performed by Agilent 2100 Bioanalyzer. The concentration of each library was quantified with KAPA Library Quantification Kit (Illumina® platforms). The high-throughput sequencing of the cDNA libraries was performed using Illumina HiSeq. 4000. The changes of expression level among samples were compared and analyzed by Ballgown. The genes classification about cell mobility, oxidative stress and lysosome were filter using the Gene Ontology (GO) database. Differentially expressed genes were identified through fold-change (FC) screening. P value < 0.05 were considered statistically meaningful.

### Cell Viability

Cell viability was determined by Cell Counting Kit-8 assay. Briefly, the MCF-7 cells were seeded into 96-well plates, six duplicate wells for each group. At the designated time, the culture medium was replaced with 100 µl medium containing 10 µl CCK-8 solution (Dojindo Molecular Technologies, Japan) and incubated at 37 °C for 2 hrs. The absorbance at 450 nm was measured using microplate reader.

### Statistical analysis

Statistical analyses were conducted using SPSS13.0 software. Data are expressed as means ± standard deviations. Statistical analyses were performed using one-way analysis of variance with post hoc Bonferroni’s test. P value < 0.05 was considered statistically significant.

## Electronic supplementary material

Figure S1, Figure S2, Figure S3, Figure S4, Figure S5
