# Supplementary material for: Residual Ammonium Persulfate in Nanoparticles Has Cytotoxic Effects on Cells through Epithelial-Mesenchymal Transition
Source: Sci Rep. 2017 Sep 18;7:11769. doi: 10.1038/s41598-017-12328-0 (PMC5603593; doi:10.1038/s41598-017-12328-0)
Supplement: Supplementary file 1 — Figure S1, Figure S2, Figure S3, Figure S4, Figure S5 [file 41598_2017_12328_MOESM1_ESM.doc]

***Supporting information***

**Residual Ammonium Persulfate in Nanoparticles Has Cytotoxic Effects on Cells Through Epithelial-Mesenchymal Transition**

*Chen Song^#^,* *Leyu Wang^#^*,* *Genlan Ye,* *Xiaoping Song,* *Yutong He, Xiaozhong Qiu**

^a^Deparment of Anatomy, Guangdong Provincial Key Laboratory of Construction and Detection in Tissue Engineering, Southern Medical University, Guangdong, Guangzhou 510515, China

# equal contribution

***To whom correnspondence should be addressed.

E-mail: [qqiuxzh@163.com](mailto:qqiuxzh@163.com), E-mail: [wangleyu889@163.com](mailto:wangleyu889@163.com)


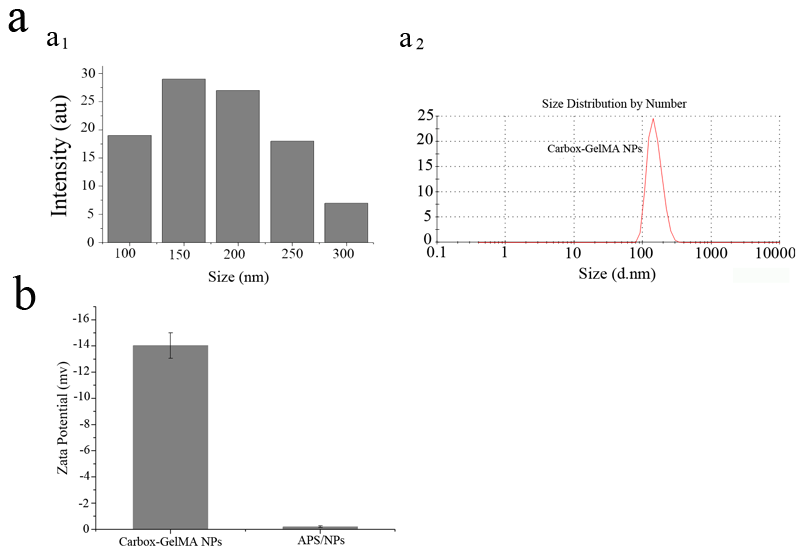


**Figure S1. Characterization of carbox-GelMA NPs.**

**(a)** The sizes distribution of carbox-GelMA NPs from more than 100 NPs (a1) and the dynamic light scattering (DLS) analysis (a2). **(b)** The surface charges of carbox-GelMA NPs and APS/ NPs.

**
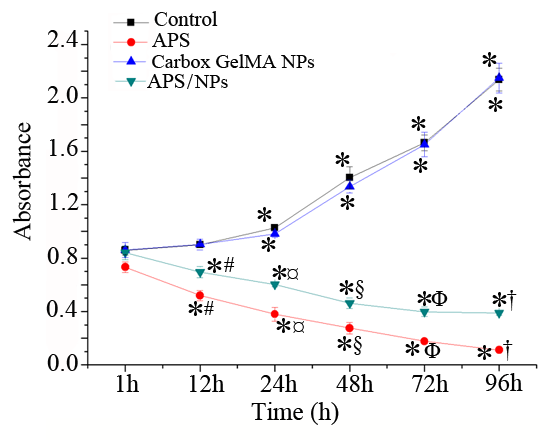
**

**Figure S2. The cell viability of MCF-7 cells in the control, APS, carbox-GelMA NPs and APS/NPs groups separately at different times.**

OD values at different time were compared with that at 1 hour in the same group (* means p < 0.05); OD values in different groups were compared with that in the control groups at the same time (#, ¤, §, Φ and † mean p < 0.05 in different groups at the same time).


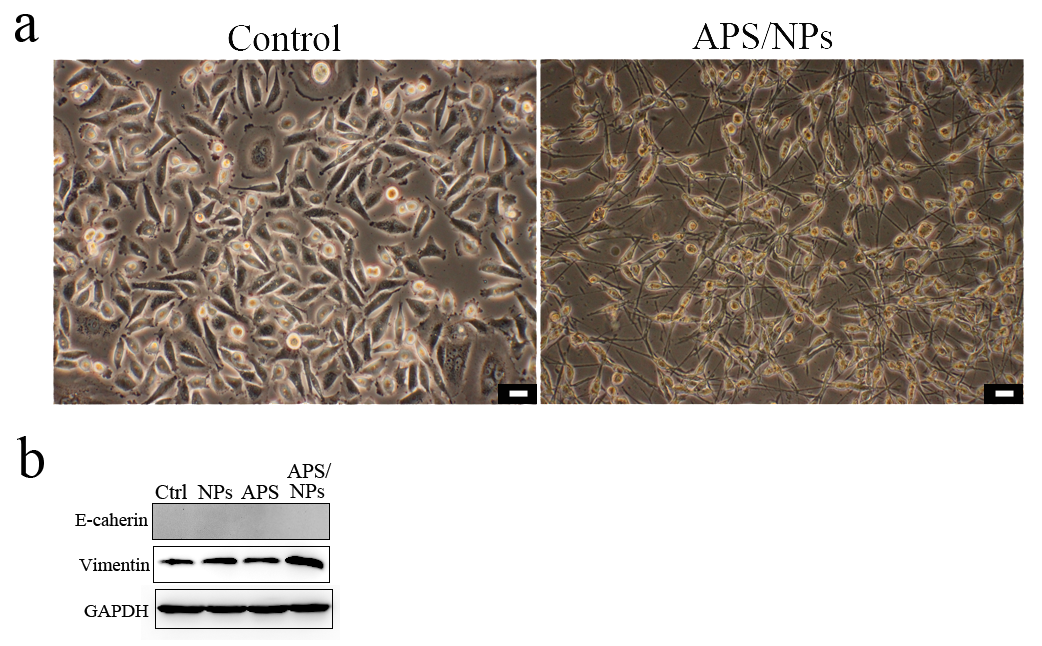


**Figure S3. The APS/NPs treatment also induced EMT in MDA-MB-231 cells.**

**(a)** After being treated with APS/NPs for 72 hrs, the MDA-MB-231 cells exhibited more fibroblast-like morphology. Scale bars: 100 µm. **(b)** The vimentin protein expression in MDA-MB-231 cells was increased after the cells were co-cultured with APS/NPs for 24 hrs.


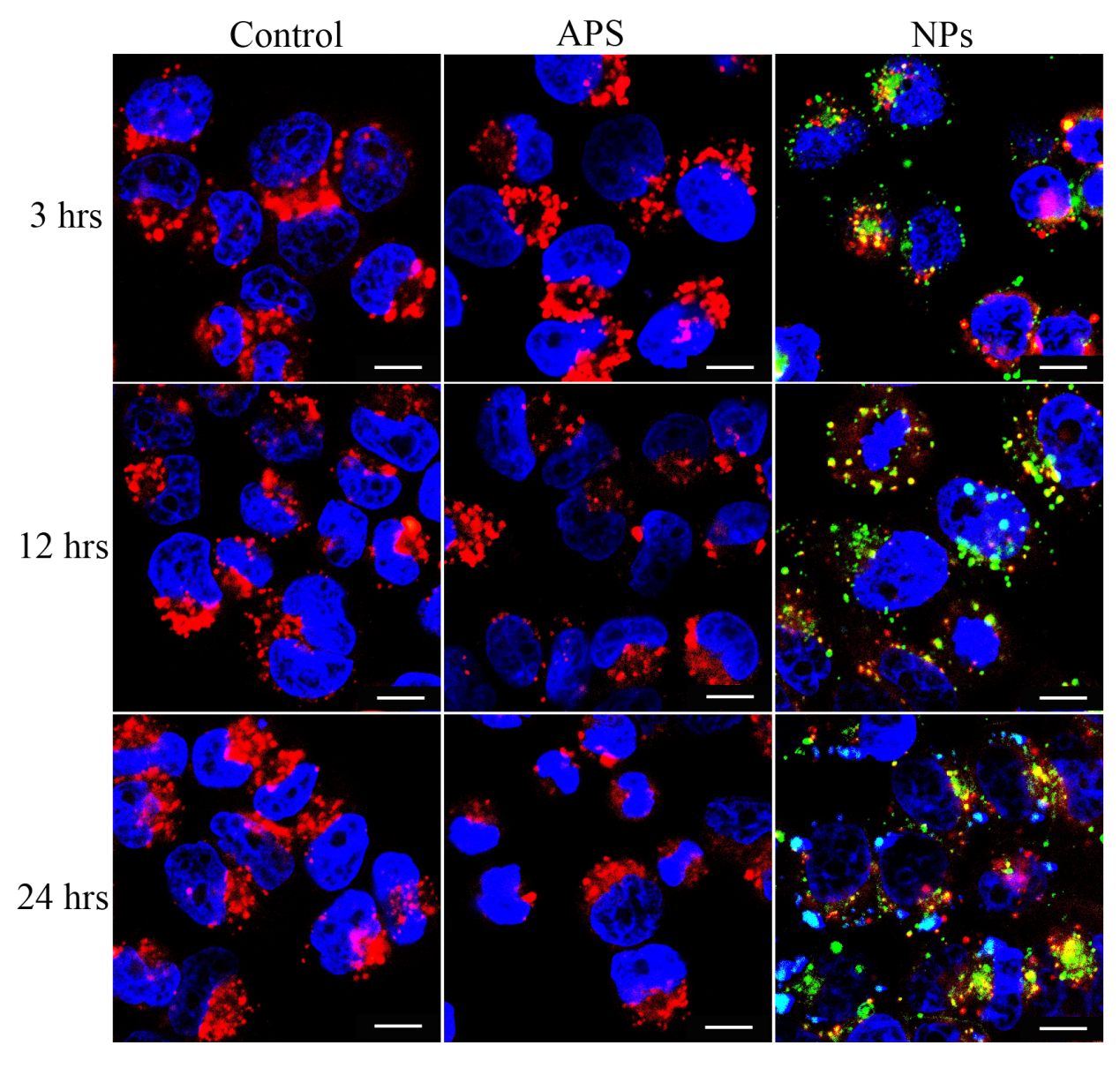


Figure S4. The lysotracker-marked lysosome (red) and FITC-labeled NPs (green) were detected by CLSM in untreated cells (control group), APS-treated cells (APS group) and NPs-treated cells (NPs group) respectively. Scale bars: 10 µm.


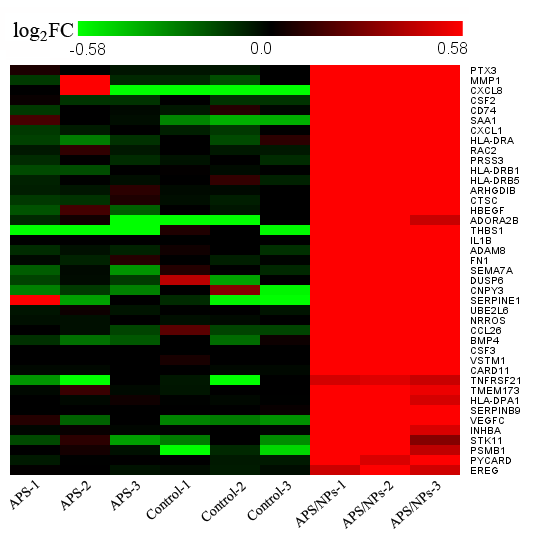


**Figure S5. The heatmap of immune response-related genes among the APS-treated MCF-7 cells, the APS/NPs-induced MCF-7-EMT cells, and the untreated MCF-7 cells.**
